# Aberrant WNT/CTNNB1 Signaling as a Therapeutic Target in Human Breast Cancer: Weighing the Evidence

**DOI:** 10.3389/fcell.2020.00025

**Published:** 2020-01-31

**Authors:** Emma H. van Schie, Renée van Amerongen

**Affiliations:** ^1^University of Amsterdam, Amsterdam, Netherlands; ^2^Section of Molecular Cytology and van Leeuwenhoek Centre for Advanced Microscopy, Swammerdam Institute for Life Sciences, University of Amsterdam, Amsterdam, Netherlands

**Keywords:** canonical Wnt signaling, non-canonical Wnt signaling, beta-catenin, breast cancer, mammary gland, stem cells, cancer stem cells

## Abstract

WNT signaling is crucial for tissue morphogenesis during development in all multicellular animals. After birth, WNT/CTNNB1 responsive stem cells are responsible for tissue homeostasis in various organs and hyperactive WNT/CTNNB1 signaling is observed in many different human cancers. The first link between WNT signaling and breast cancer was established almost 40 years ago, when *Wnt1* was identified as a proto-oncogene capable of driving mammary tumor formation in mice. Since that discovery, there has been a dedicated search for aberrant WNT signaling in human breast cancer. However, much debate and controversy persist regarding the importance of WNT signaling for the initiation, progression or maintenance of different breast cancer subtypes. As the first drugs designed to block functional WNT signaling have entered clinical trials, many questions about the role of aberrant WNT signaling in human breast cancer remain. Here, we discuss three major research gaps in this area. First, we still lack a basic understanding of the function of WNT signaling in normal human breast development and physiology. Second, the overall extent and precise effect of (epi)genetic changes affecting the WNT pathway in different breast cancer subtypes are still unknown. Which underlying molecular and cell biological mechanisms are disrupted as a result also awaits further scrutiny. Third, we survey the current status of targeted therapeutics that are aimed at interfering with the WNT pathway in breast cancer patients and highlight the importance and complexity of selecting the subset of patients that may benefit from treatment.

## Introduction

WNT proteins and their downstream effectors form a highly conserved signaling network that regulates tissue morphogenesis during development and adult tissue homeostasis in virtually all multicellular animals studied to date (van Amerongen and Nusse, 2009; Loh et al., 2016; Schenkelaars et al., 2017). The mammalian genome contains 19 *WNT* genes, encoding 19 different WNT proteins. These can bind and activate 10 different FZD receptors and a handful of co-receptors, thereby initiating different intracellular signaling cascades. ‘Canonical’ WNT signaling is defined by its use of β-catenin (CTNNB1) as main downstream effector and transcriptional co-activator of TCF/LEF target gene expression (MacDonald et al., 2009; Clevers and Nusse, 2012; Nusse and Clevers, 2017). ‘Non-canonical’ WNT signaling responses do not use CTNNB1, but instead activate different signaling molecules with profound impact on the cytoskeleton and cell migration (Komiya and Habas, 2008; van Amerongen, 2012; VanderVorst et al., 2018).

For both historic and experimental reasons, the intestinal epithelium has become the benchmark against which all other tissues are weighed when it comes to WNT signaling. This has shaped both our thinking and our terminology, with the intestine frequently being referred to as the “typical” example. A large body of literature shows that stem cell self-renewal and differentiation in the intestine and other endodermal derivatives is critically dependent on WNT/CTNNB1 signaling (Sato et al., 2009; Barker et al., 2010; Huch et al., 2013a, b; Clevers et al., 2014; Clevers, 2016). Hyperactive WNT/CTNNB1 signaling is a hallmark of colorectal cancer, both in early stages of polyp formation and at later stages of invasion and metastasis (Zhang and Shay, 2017). In this context, increased WNT/CTNNB1 signaling mainly results from genetic mutations in the *APC* gene, which encodes a negative regulator of CTNNB1 (Fodde, 2002). The unambiguous genetic evidence from human tumors leaves little doubt about the relevance of aberrant WNT/CTNNB1 signaling in the initiation and progression of colorectal cancer.

The involvement of WNT signaling in breast cancer remains less well understood (Yu et al., 2016; Alexander, 2018). This is surprising, given that the link between WNT signaling and breast cancer is as old as the WNT research field itself (Nusse and Varmus, 2012). In fact, the first mammalian WNT gene (*Wnt1*, originally identified as *int-1*) was discovered as a proto-oncogene capable of driving mammary tumor formation in mice (Nusse and Varmus, 1982). Here we review the evidence, highlight current research gaps and indicate future avenues worth exploring to dissect the role of WNT signaling in human breast cancer.

## How Important Is WNT Signaling for Development and Maintenance of the Human Breast?

A first major knowledge gap is our lack of a basic understanding of the role of WNT signaling in human breast development and physiology. The mammary gland largely develops after birth and undergoes dynamic tissue remodeling throughout life. The most prominent changes occur in puberty (when the breast tissue develops under the influence of rising levels of estrogen and progesterone), and during pregnancy and lactation (when it differentiates and produces milk to nurture the offspring). Given how critical this tissue has been for our survival as a mammalian species and in view of the prevalence and mortality of breast cancer across different societies in women worldwide, it remains somewhat strange that we still have an incomplete picture of the molecular, cell and tissue biology of the human breast. In fact, one of the most detailed studies of human breast development, and individual variation therein, arguably dates back to 1840^1^.

Most of what we know about WNT signaling in mammary gland biology and breast cancer comes from studies in mice, where both CTNNB1-dependent and -independent signaling are essential for mammary gland development, branching morphogenesis and function during embryogenesis and in postnatal life (Brisken et al., 2000; Chu et al., 2004; Veltmaat et al., 2004; Badders et al., 2009; Roarty et al., 2015; Yu et al., 2016). The mouse was discovered as a useful organism for studying the link between hormones and breast cancer well over a century ago (Lathrop and Loeb, 1916), but it really came to the fore as an experimental model system with the discovery of the fat pad transplantation assay (Deome et al., 1959). This technique remains indispensable for studying the growth, differentiation and regenerative properties of different mammary epithelial cell populations (Faraldo et al., 2015; Wronski et al., 2015). Nowadays, robust protocols allow the prospective isolation of mammary stem cells (capable of forming a new epithelial network upon transplantation) via fluorescence activated cell-sorting (FACS) (Shackleton et al., 2006; Stingl et al., 2006; Prater et al., 2013; Gao et al., 2016). More recently, genetically engineered mouse models have allowed sophisticated lineage tracing approaches, which have been instrumental for studying mammary stem and progenitor cell behavior *in situ* (van Amerongen, 2015; van de Moosdijk et al., 2017).

Multiple efforts have been made to delineate the mouse mammary epithelial cell hierarchy. The cumulative lineage tracing literature suggests that postnatal mammary gland development, homeostasis and remodeling are mainly driven by unipotent basal and luminal stem cells (Van Keymeulen et al., 2011; Davis et al., 2016; Wuidart et al., 2016, 2018; Scheele et al., 2017), although a rare fraction of bipotent stem cells likely co-exists (Wang et al., 2015). At least some mammary stem cells are WNT/CTNNB1 responsive (Zeng and Nusse, 2010; De Visser et al., 2012; van Amerongen et al., 2012a; Plaks et al., 2013; Wang et al., 2015; Blaas et al., 2016). However, this does not automatically imply that homeostasis and remodeling of the mammary epithelium is as strictly controlled by WNT/CTNNB1 responsive stem cells as appears to be the case for the intestinal epithelium. Moreover, stem cell plasticity can be induced by transplantation (Van Keymeulen et al., 2011; van Amerongen et al., 2012a) or oncogenic mutations (Koren et al., 2015; Van Keymeulen et al., 2015), raising the question if mammary stem and progenitor cells should be forced into a rigid hierarchy to begin with.

How findings from the mouse translate to the human breast remains unclear. In both human and mouse, the mammary gland is comprised of a non-stereotypically branched, ductal network composed of a bilayer of basal and luminal epithelial cells. Yet neither the two tissues, nor the experimental systems available to study each of them, are directly comparable between the two species. Major differences exist in the composition of the stroma, with the mouse mammary gland containing a higher proportion of adipocytes (hence the name ‘fat pad’ for the stromal pocket into which cells can be transplanted) and the human breast containing considerably more collagen. This constitutes a different molecular signaling environment with very different mechanobiological properties. Breast tissue composition changes throughout life and varies between individual women (Sun et al., 2014). Prominent differences in the expression pattern of epithelial cell markers between mouse and human also exist, although these are frequently ignored. For example, KRT14 reliably marks basal cells in the mouse mammary gland but is also expressed in a fraction of luminal cells in the human breast (Santagata et al., 2014; Dontu and Ince, 2015; McNally and Stein, 2017; Gerdur Ísberg et al., 2019).

Unlike in mice, human stem cell activity cannot be readily visualized *in vivo*. Unraveling the stem and progenitor cell hierarchy in the breast has thus proven difficult, but a recent study managed to use Cytochrome C Oxidase deficiency to identify multi-lineage differentiation in the healthy breast, presumably from stem cells in the luminal layer (Cereser et al., 2018). Experimental systems to study self-renewal and differentiation of human breast epithelial cells are limited to *in vitro* cell culture assays. Primary mammosphere cultures (in which cells are grown in suspension to enrich for cells with self-renewal properties) are frequently used to evaluate human breast stem cell activity (Shaw et al., 2012). However, this link is indirect and may not reflect the *in vivo* situation.

Access to healthy human breast tissue for experimental purposes is usually restricted to the leftover material from breast reduction surgeries. FACS protocols have been developed to isolate different cell populations from these specimens, including an ALDH + population with stem/progenitor cell properties as evaluated by multi-lineage differentiation in a 2D clonogenic colony formation assay (Ginestier et al., 2007). Transcriptional profiling of these cells revealed that they express high levels of *WNT2* and *RSPO3*, suggesting an autocrine source of ligands and agonists (Colacino et al., 2018). Mammosphere cultures are typically maintained in the absence of exogenous WNT proteins, but cells in these cultures do express *FZD2* (Shaw et al., 2012). Although primary human mammosphere cultures appear to be relatively insensitive to DKK1-mediated inhibition of WNT signaling (Lamb et al., 2013), multiple *WNT* genes can be induced in these cultures upon stimulation with estrogen or progesterone (Arendt et al., 2014). Comparative transcriptional profiling between mouse and human epithelial cells suggests that active WNT/CTNNB1 signaling in the basal cell population is conserved between the two species (Lim et al., 2010) and long-term maintenance of primary human as well as mouse mammary epithelial cells in Matrigel has been reported in the presence of WNT3A-containing media (Zeng and Nusse, 2010; Sachs et al., 2018).

Summarizing, the human breast likely also uses WNT signaling for growth and differentiation. However, the WNT-secreting and WNT-responsive cells have not been clearly demarcated. Single cell RNA sequencing studies will likely shed more light on the stem and progenitor cell hierarchy in the healthy human breast, and on the position of WNT/CTNNB1 signaling in this hierarchy, in the foreseeable future (Holliday and Speirs, 2011). If and how CTNNB1-dependent and –independent signaling functionally controls proliferation, differentiation and branching morphogenesis of primary human breast epithelial cells is something that can likely only be answered using primary 3D organotypic cultures (Linnemann et al., 2015, 2017).

## Is WNT Signaling Deregulated in Human Breast Cancers?

A second research gap is the lack of specific markers to reliably measure WNT signaling activity in human breast cancer. CTNNB1-independent signaling responses are notorious for their lack of robust readouts in most mammalian cells and tissues. For CTNNB1-dependent signaling, such readouts are available: Reporter constructs with concatemerized TCF/LEF binding sites can be introduced into cells and patient derived xenografts to measure WNT/CTNNB1 signaling (Green et al., 2013; Many and Brown, 2014). However, this approach is unsuitable for monitoring pathway activity in histological specimens, nor does it probe multifactorial signaling in the endogenous chromatin context (Nakamura et al., 2016; Doumpas et al., 2019).

Two of the earliest described WNT/CTNNB1 target genes are *CCND1* and *MYC* (He et al., 1998; Shtutman et al., 1999). Elevated protein levels of CCND1 and MYC are detected in a high proportion of invasive ductal breast carcinomas, but this does not always correlate to CTNNB1 expression levels (Wong et al., 2002; He et al., 2014). Given their general involvement in cell proliferation, upregulation of *CCND1* and *MYC* can be achieved in myriad ways (Lindqvist et al., 2014). So far, *AXIN2* appears to be one of the few universal target genes that could be used to reliably measure relative WNT/CTNNB1 signaling activity in human breast cancer (Lustig et al., 2001; Jho et al., 2002).

In the absence of a well-defined, mammary-specific WNT/CTNNB1 target gene expression program and given the preponderance of paraffin embedded tumor specimens, immunohistochemical detection of CTNNB1 protein levels has been used as the most direct way to readout WNT/CTNNB1 signaling. From these analyses it has been known for a long time that elevated intracellular levels of CTNNB1, a hallmark of active WNT/CTNNB1 signaling, can be detected by immunohistochemistry in a significant (13–77%) proportion of all ductal and lobular breast cancer samples (Jonsson et al., 2000; Karayiannakis et al., 2001; Wong et al., 2002; Ozaki et al., 2005; Prasad et al., 2008a; He et al., 2014; Hou et al., 2018). Care should be taken when performing and interpreting these experiments: Dogma dictates that active WNT/CTNNB1 signaling results in increased nuclear CTNNB1 levels, but those with more hands on experience in the field know that changes in CTNNB1 can be quite subtle and even modest (2–5 fold) increases in the levels of intracellular CTNNB1 can be more than sufficient to robustly activate TCF/LEF target gene expression (Jacobsen et al., 2016).

**FIGURE 1:**
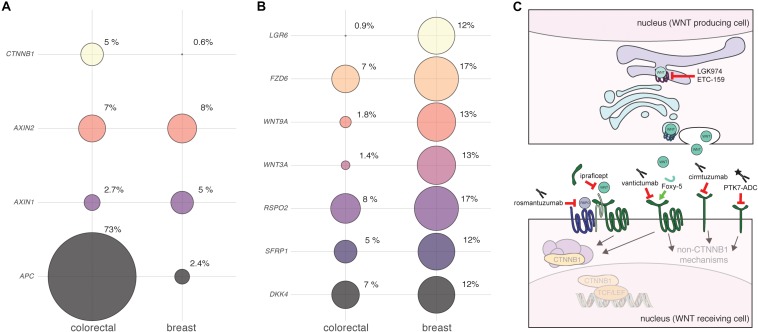
Detecting and targeting aberrant WNT signaling in human breast cancer. **(A,B)** Bubble plots illustrating the alteration of different WNT pathway components in breast versus colorectal cancer. Plots were generated using data from http://cbioportal.org (accessed on 20 September 2019), using the following datasets: Colorectal Adenocarcinoma (TCGA, Provisional), samples with mutation and copy number alteration data (220 patients/samples). Breast Invasive Carcinoma (TCGA, Provisional), samples with mutation and copy number alteration data (963 patients/samples). Circle sizes reflect the proportion of samples with alterations in each of the genes depicted, with the actual percentages shown. Note that copy number alterations (amplifications + deletions) and mutations (truncations + substitutions) were combined into a single score. No distinction was made between breast cancer subtypes. Data were not corrected for overall differences in mutation rates or genome instability between the different tumor types. No inference can be made about RNA and protein expression level changes based solely on these analyses. **(A)** *APC* is the most prominently mutated gene in colorectal cancer. Other endodermal cancers, including hepatocellular carcinoma, also show frequent genetic mutations in WNT/CTNNB1 signaling components (White et al., 2012). Depending on the tissue of origin and tumor subtype, activating mutations in *CTNNB1* itself or inactivating mutations in negative regulators like *APC* or *AXIN1* are more or less prevalent (Yanagisawa et al., 1992; Morin, 1997; Ishizaki et al., 2004). In breast cancer, genetic mutations in *APC* are rare. However, epigenetic changes such as *APC* promoter hypermethylation have been reported in the literature, with the highest incidence observed in inflammatory breast cancer (Jin et al., 2001; Van Der Auwera et al., 2008; Lindqvist et al., 2014). **(B)** The top genes that show genetic alterations in breast cancer are implicated to a lesser extent in colorectal cancer. Note that all of these components function at the level of ligand and receptor binding. The top two hits, *RSPO2* and *FZD6*, have both been linked to reduced metastasis free survival, but likely operate via different WNT signaling mechanisms (Corda et al., 2017; Coussy et al., 2017). It should be stressed that in this respect, breast cancer is not unique. As more and more genome-wide expression profiling studies are becoming available, evidence is accumulating that many different cancers likely display changes in WNT/CTNNB1 signaling in the absence of mutations in *APC* or *CTNNB1* (Wiese et al., 2018; Flanagan et al., 2019b). In addition, it was recently demonstrated that FZD7, which functions upstream of APC and CTNNB1, is required for WNT/CTNNB1 signaling in gastric tumors irrespective of their *APC* status (Flanagan et al., 2019a). This is reminiscent of earlier studies hinting toward a similar phenomenon for other upstream components (Suzuki et al., 2004). Even in colorectal cancer, the situation may thus be far more complex than envisioned, and the local niche may continue to affect signaling levels even when the WNT/CTNNB1 pathway is intrinsically activated through genetic mutations in *APC* (van Neerven and Vermeulen, 2019). **(C)** Cartoon showing the points of interception for WNT-pathway targeting drugs that are currently in clinical trials. See text for details.

Clinical evidence suggests that WNT/CTNNB1 signaling is elevated across multiple subtypes of human breast cancer. Aggressive triple negative breast carcinomas (TNBC) were found to be enriched for elevated CTNNB1 levels compared to luminal A, luminal B or HER2+ tumors (Khramtsov et al., 2010). Higher levels of intracellular CTNNB1 are associated with a higher tumor grade (Sormunen et al., 1999) and poor prognosis (Lin et al., 2000; Khramtsov et al., 2010). The highest levels of CTNNB1 are found in metaplastic carcinomas and non-metastasizing fibromatosis – two rare subsets of breast cancer (Lacroix-Triki et al., 2010). Here, up to 90% of tumors show increased levels of CTNNB1 and a proportion of these may contain activating genetic mutations in the *CTNNB1* gene (Abraham et al., 2002; Hayes et al., 2008; Hennessy et al., 2009). For the most part however, and unlike the situation encountered in colorectal cancer, genetic mutations in *APC*, *AXIN* or *CTNNB1* are virtually non-existent in human breast tumors (Figure 1A). As first proposed many years ago, this discrepancy can likely be explained by tissue-specific differences in sensitivity to WNT/CTNNB1 signaling (Gaspar and Fodde, 2004; Gaspar et al., 2009).

In the absence of any apparent genetic mutations, what then is the cause of elevated CTNNB1 levels in human breast cancer? In the normal human breast, CTNNB1 is mainly detected in the cell membrane as part of adherens junctions (Hashizume et al., 1996). It cannot be excluded that the increase in CTNNB1 could therefore, at least partially, be due to its release from these junctions upon loss of CDH1, given that this is a frequent event in more advanced and invasive tumors (Prasad et al., 2008b; Zeljko et al., 2011). However, another possibility is that CTNNB1 levels are increased as a direct result of enhanced WNT/CTNNB1 signaling due to changes in the expression levels of upstream WNT pathway components. In large public breast cancer datasets, changes at the level of ligands, (ant)agonists and receptors are readily apparent (Figure 1B). Moreover, the cumulative literature provides ample evidence of changes in the levels of ligands and receptors in primary or metastatic human breast cancer (Table 1). In interpreting these findings, some caution is warranted. First, few of the RNA expression level changes have been shown to affect protein levels. Second, where such follow up is performed, antibody specificity has not always been properly validated.

**TABLE 1:** Comprehensive overview of ligand (*WNT1-16)* and receptor (*FZD1-10, LRP5-6, ROR1-2, RYK, PTK)* genes and their implication in human breast cancer based on a survey of the primary literature.

Gene	Mechanism*		Drug**	Gene expression changes detected at the level of		Reference
				
	*CTNNB1*	*other*		*RNA*	*protein*	
*WNT1*	X	?	PORCN_i_	0		Corda et al., 2017
				0		Milovanovic et al., 2004
				0		Watanabe et al., 2004
				+		Ayyanan et al., 2006
				+		Ain et al., 2011
					+	Wong et al., 2002
*WNT2*	X	?	PORCN_i_	+		Dale et al., 1996
				+		Ellsworth et al., 2009
				+		Huguet et al., 1994
				+		Katoh, 2001
				+		Watanabe et al., 2004
*WNT2B*	X	?	PORCN_i_	n.a.	n.a.	n.a.
*WNT3*	X	?	PORCN_i_	0		Huguet et al., 1994
*WNT3A*	X	?	PORCN_i_	n.d.		Huguet et al., 1994
				0		Corda et al., 2017
*WNT4*	X	X	PORCN_i_	+		Ayyanan et al., 2006
				+		Huguet et al., 1994
				+		Tsai et al., 2015
*WNT5A*	X	X	PORCN_i_ Foxy-5	–	–	Borcherding et al., 2015
					–	Dejmek et al., 2005
					–	Jönsson et al., 2002
				–		Martin et al., 2005
				–		Trifa et al., 2013
					–	Zhong et al., 2016
				+		Iozzo et al., 1995
				+		Lejeune et al., 1995
*WNT5B*	X	?	PORCN_i_	+		Corda et al., 2017
				+		Klemm et al., 2011
*WNT6*	X	?	PORCN_i_	0		Milovanovic et al., 2004
				+		Ain et al., 2011
*WNT7A*	X	?	PORCN_i_	n.d.		Huguet et al., 1994
				+		Avgustinova et al., 2016
				–		Yi et al., 2017
*WNT7B*	X	?	PORCN_i_	–		Milovanovic et al., 2004
				+		Huguet et al., 1994
				+		Yeo et al., 2014
*WNT8A*	?	?	PORCN_i_	n.a.	n.a.	n.a.
*WNT8B*	?	?	PORCN_i_	n.a.	n.a.	n.a.
*WNT9A*	?	?	PORCN_i_	n.a.	n.a.	n.a.
*WNT9B*	?	?	PORCN_i_	n.a.	n.a.	n.a.
*WNT10A*	X	?	PORCN_i_	–		Ain et al., 2011
*WNT10B*	X	?	PORCN_i_	+		Bui et al., 1997
					+	Wend et al., 2013
*WNT11*	?	X	PORCN_i_	+		Corda et al., 2017
*WNT16*	?	?	PORCN_i_	n.a.	n.a.	n.a.
*FZD1*	?	?	OMP18R5 (vantictumab)	+		Milovanovic et al., 2004
*FZD2*	?	?	OMP18R5 (vantictumab)	+		Gujral et al., 2014
				+		Milovanovic et al., 2004
*FZD3*	?	?		+		Bell et al., 2017
*FZD4*	X	?		n.a.	n.a.	n.a.
*FZD5*	X	?	OMP18R5 (vantictumab)	n.a.	n.a.	n.a.
*FZD6*	?	X		+	+	Corda et al., 2017
*FZD7*	X	?	OMP18R5 (vantictumab)	+		Chakrabarti et al., 2014
				+		Dey et al., 2013
				+		Jia et al., 2018
				+		Yang et al., 2011
*FZD8*	X	?	OMP18R5 (vantictumab)		+	Jiang et al., 2016
			OMP-54F28 (ipafricept)	–		Wang et al., 2012
*FZD9*	?	?		C^M^pG		Conway et al., 2014
*FZD10*	?	?		0		de Groot et al., 2014
*LRP5*	X	–		n.a.	n.a.	n.a.
*LRP6*	X	–		+		Lindvall et al., 2009
				+		Liu et al., 2010
				_		Ma et al., 2017
*ROR1*	?	X	Cirmtuzumab		+	Balakrishnan et al., 2017
					+	Cao et al., 2018
					+	Chien et al., 2016
					+	Cui et al., 2013
					+	Zhang et al., 2012
*ROR2*	?	X			–	Li et al., 2014
					+	Henry et al., 2015
*RYK*	?	?		–		Borcherding et al., 2015
*PTK7*	?	?	PTK7-ADC	+		Ataseven et al., 2013
				+		Damelin et al., 2017
				+		Gärtner et al., 2014

Only data collected from freshly isolated tumors (e.g., microarrays, qRT-PCR, Western blotting) or fixed tumor samples (e.g., immunohistochemistry) were used. Data obtained from experiments on established human breast cancer cell lines or patient-derived xenografts were not included. Subtype-specific differences have been incompletely investigated, partially due to small cohort sizes. As an example, when all breast cancer subtypes were grouped together, 75% scored negative for WNT10B protein expression (Wend et al., 2013), corresponding to an earlier finding at the RNA level (Bui et al., 1997). However, 90% of TNBC samples scored positive (Wend et al., 2013). Similarly, *FZD9* shows more frequent hypermethylation in hormone-receptor positive invasive breast cancers compared to those that are scored as hormone-receptor negative, as well is in those tumors that have a wildtype as opposed to a mutant *TP53* status (Conway et al., 2014). *Potential signaling mechanism based on evidence from the cumulative Wnt literature supporting involvement of the gene product in WNT/CTNNB1 signaling and/or non-canonical (other) signaling events. **Potential target for the indicated drugs based on substrate specificity of the listed therapeutics described in the literature. –, Lower RNA or protein expression detected in primary breast cancer tissue compared to normal tissue and/or lower expression is associated with worse prognosis. 0, similar expression in breast cancer tissue and normal tissue. +, Higher RNA or protein expression detected in primary in breast cancer tissue compared to normal tissue and/or higher expression is associated with worse prognosis. n.a., no data available. n.d., tested, but not detectable. PORCN_*i*_, PORCN inhibitors. C^*M*^pG, DNA methylation detected.

Since absolutely no inference about cell biological mechanisms can be made solely based on expression level changes, functional follow up is crucial to determine the implications of these alterations. For example, only FZD7 is consistently found to signal through CTNNB1/TCF in human breast cancer cells, thereby affecting cell proliferation (Yang et al., 2011; Chakrabarti et al., 2014; Riley and Day, 2017). In contrast, copy number gain of the *FZD6* gene, which can be readily detected in human breast cancer cohorts (Figure 1B) and most predominantly in TNBC, most likely exerts its effects on cell motility and invasion via alternative, non-canonical WNT signaling mechanisms (Corda et al., 2017). For other components, such as *RSPO2*, *RSPO4* and to a lesser extent *LGR5* and *LGR6*, the overexpression of which is enriched in TNBC, the mechanism is more likely to involve amplification of the WNT/CTNNB1 signaling response (Coussy et al., 2017). Importantly, the separation between canonical and non-canonical WNT signaling is not black and white. For instance, WNT5A, still frequently regarded as the “typical” non-canonical WNT ligand, can both repress and activate CTNNB1-dependent signaling, *in vitro* as well as *in vivo* (Mikels and Nusse, 2006; van Amerongen et al., 2012b). Especially in the context of cancer, where cellular signaling pathways are invariably deregulated, unexpected signaling activities are likely to be encountered (Grossmann et al., 2013).

Summarizing, more extensive transcriptional and epigenetic profiling of tumor and adjacent normal tissue is needed to reveal the true extent of aberrant WNT signaling in human breast cancer. Early studies reported hypermethylation, and presumably silencing, of genes encoding secreted WNT-pathway inhibitors as a potential mechanism for disrupting the balance in WNT signaling in breast cancer. Examples are widespread and include *WIF1* (Wissman et al., 2003; Ai et al., 2006; Veeck et al., 2009), *SFRP1* (Ugolini et al., 2001; Veeck et al., 2006; Suzuki et al., 2008), *SFRP2* (Suzuki et al., 2008; Lindqvist et al., 2014), *SFRP5* (Suzuki et al., 2008; Veeck et al., 2008a; Lindqvist et al., 2014), *DKK1* (Forget et al., 2007; Suzuki et al., 2008) and *DKK3* (Veeck et al., 2009; Lindqvist et al., 2014; Yamaguchi et al., 2015). Epigenetic analyses, such as those measuring DNA methylation levels, are now becoming part of the standard work flow for large consortia. The first of such analyses indeed revealed extensive changes in WNT signaling components across breast tumors (Koval and Katanaev, 2018). The main challenge still lies ahead as we face the daunting task of properly interpreting these experimental findings. For instance, *DKK3* and *WIF1* methylation was detected in a similar proportion of breast cancer patients, but only *DKK3* methylation was a prognostic marker of survival (Veeck et al., 2009). And while one study reported *SFRP2* promoter hypermethylation in more than 80% of breast cancer patients (Veeck et al., 2008b), a recent report suggests that, in contrast, elevated serum levels of SFRP2 may serve as an independent marker for poor prognosis (Huang et al., 2019). Future studies will also have to focus on subtype-specific differences.

## Will Breast Cancer Patients Benefit From Drugs Targeting the WNT Pathway?

Our current lack of understanding which patients are most likely to benefit from treatment with WNT inhibitors is a third major knowledge gap. Several drugs that interfere with the WNT signaling pathway are currently being tested in clinical trials (for recent reviews see Krishnamurthy and Kurzrock, 2018; Ghosh et al., 2019). After decades of ill-fated attempts to block WNT signaling downstream of CTNNB1, the current developmental pipeline is fueled by two different rationales (Figure 1C). The first is the conceptual notion that, even in the absence of apparent mutations, WNT/CTNNB1 plays a central role in the maintenance of multiple adult tissue stem cell populations and, by analogy and extension, in cancer stem cells. This line of reasoning forms the basis for the development of drugs that inhibit WNT protein secretion, such as the PORCN inhibitors LGK974 and ETC-159 (Liu et al., 2013; Madan et al., 2016). The main adverse effects reported for PORCN inhibitors in Phase I clinical trials are related to loss of bone density (Ng et al., 2017; Tan et al., 2018). Somewhat surprisingly, the systemic toxicity of PORCN inhibitors appears to be relatively limited. One potential explanation for this observation comes from experiments conducted in mice. Here, the WNT-secreting intestinal myofibroblasts, which constitute the intestinal stem cell niche, were shown to be intrinsically resistant to xenobiotics, including PORCN inhibitors, because they express a subset of multidrug efflux pumps (Chee et al., 2018). While this opens a therapeutic window, it also leads to the sobering conclusion that tumor cells may likely evolve similar resistance mechanisms upon prolonged treatment. In fact, these same ATP-binding cassette (ABC) transporters have long been implicated in acquired multidrug resistance in cancer, albeit in the context of classical chemotherapeutic agents rather than targeted therapeutics (Robey et al., 2018). In addition, although it is generally assumed that all WNT ligands require PORCN for their secretion, exceptions to this rule may exist (Rao et al., 2018).

The second rationale for designing drugs that interfere with WNT signaling are more focused and evidence based. These efforts are directed toward specific WNT-pathway components that show altered expression in human tumors. Examples include the anti-RSPO3 antibody OMP-131R10/rosmantuzumab and the decoy receptor FZD8-CRD OMP-54F28/ipafricept (Cattaruzza et al., 2015; Le et al., 2015). So far, the most promising results for breast cancer have been obtained with the broad-spectrum anti-FZD antibody OMP-18R5/vantictumab, which blocks FZD1, 2, 5, 7, and 8 (Gurney et al., 2012). In pre-clinical trials, OMP-18R5 was shown to inhibit the outgrowth of patient derived breast cancer xenografts, thus demonstrating potential efficacy against breast cancer (Gurney et al., 2012; Fischer et al., 2017). A phase Ib clinical trial in HER2^–^ breast cancer patients identified a four-gene signature (*FBXW2*, *CCND2*, *CTBP2*, and *WIF1*) as a potential predictive biomarker for the response to combined treatment with paclitaxel and vantictumab (Zhang et al., 2018). Structure guided design will likely help in generating more specific antibodies that target individual FZD receptors (Raman et al., 2019). Based on the available data, FZD6 and FZD7 seem obvious candidates for therapeutic intervention (Figure 1 and Table 1).

Few WNT-pathway targeting drugs that are currently in clinical trials were explicitly developed with breast cancer in mind. A notable exception is Foxy-5, a peptide mimetic of WNT5A that was designed with the goal of blocking breast cancer metastasis by reconstituting a – presumably non-CTNNB1 driven –WNT5A signaling response in cancers that had lost *WNT5A* expression (Säfholm et al., 2008). While WNT5A protein expression was found to be low in 75% of TNBC tumors, medium to high expression was detected in 75% of ER+ breast cancer samples (Borcherding et al., 2015). Furthermore, expression levels may change upon treatment, as WNT5A protein levels were significantly higher in 79% of patients after relapse and elevated WNT5A levels were also associated with the induction of multidrug resistance (Hung et al., 2014).

In many cancers, including breast cancer, only a small population of tumor cells, the so-called ‘cancer stem cells,’ may be responsible for driving tumor growth. Human breast cancer stem cells were first identified as tumor initiating cells following transplantation into immunocompromised mice (Al-Hajj et al., 2003) and have been connected to metastasis formation and resistance to therapy. Given the presumed importance of WNT/CTNNB1 signaling in breast cancer stem cell maintenance (Lamb et al., 2013; Jang et al., 2015; Hou et al., 2018), it is somewhat counterintuitive that the non-canonical co-receptor ROR1 is emerging as a potential key mediator of chemoresistance in breast cancer stem cells (Zhang et al., 2019). Overexpression of ROR1 is a prognostic marker in TNBC (Chien et al., 2016) and the anti-ROR1 antibody cirmtuzumab, originally developed for treating chronic lymphocytic B-cell leukemia (Zhang et al., 2013), is therefore also in clinical trials for human breast cancer. Initial interest in ROR1 as a potential therapeutic target arose because of its low expression in healthy adult tissues, although a new antibody against ROR1, specifically designed for immunohistochemistry on FFPE samples, shows higher endogenous ROR1 expression than previously suspected (Shabani et al., 2015; Balakrishnan et al., 2017). Another unexpected candidate for targeting breast cancer stem cells surfaced in the form of PTK7, a WNT receptor whose function is not yet completely elucidated (Damelin et al., 2017). PTK7-ADC, a PTK7-targeting antibody that is conjugated to a cytotoxic drug, has also entered phase I clinical trials for metastatic TNBC (Radovich et al., 2019).

Summarizing, it is still too early to conclude anything about the impact of these drugs on breast cancer patient survival. If these therapeutics continue on to more advanced stages of clinical testing, the main challenge will still be to demonstrate true clinical efficacy by rationally selecting those patients that are most likely to benefit from treatment.

## Discussion

The absence of well-defined genetic mutations complicates our assessment of the functional importance of aberrant WNT signaling in human breast cancer. No definitive or generalized conclusions can be drawn about the role of either WNT/CTNNB1 or CTNNB1-independent WNT signaling at this point. Given their pleiotropic effects, we need a lot more insight into how these different signal transduction routes affect breast cancer initiation and progression. For this, we need to unravel the basic biological mechanisms through which the complex WNT signaling network controls normal human breast development and physiology. These studies will do more than just satisfy scientific curiosity: They will ultimately be critical to determine which breast cancer subtypes or individual patients are most likely to benefit from targeted therapeutics designed to interfere with WNT signaling activity, taking into account the growth promoting and inhibitory activities of individual ligand/receptor pairings in different cellular contexts.

Both patient selection and monitoring of their clinical response will require new assays and biomarkers. Our drug intervention strategies, in turn, need to be fine-tuned in such a way that individual WNT/receptor interactions or downstream signaling responses can be blocked or activated with great precision. For instance, whereas downregulation of DKK1 has been linked to lung metastases, patients with high levels of DKK1 more frequently present with bone metastases (Zhuang et al., 2017). And while the former has been suggested to occur via a non-canonical signaling mechanism, the latter likely occurs through DKK1-mediated inhibition of WNT/CTNNB1 signaling. In either case, the use of a PORCN inhibitor or a pan-FZD antibody would seem ill advised in both of these cases. Moreover, the adverse effects of these pan-WNT inhibitors on bone density will need to be overcome to advance their clinical use (Madan et al., 2018).

Finally, breast cancer is a systemic disease and the involvement of WNT signaling should be considered from this perspective as well. Both in mice and humans, loss of *TP53* has recently been associated with the induction of WNT protein production, which may in turn stimulate the immune system to promote metastasis (Kim et al., 2019; Liu et al., 2019; Wellenstein et al., 2019). Likewise, cytokine signaling from the local bone microenvironment may promote metastatic colonization by initiating an autocrine WNT signaling loop in human breast cancer stem cells (Eyre et al., 2019). At present, functional studies almost invariably fall back on the use of established human breast cancer cell lines. It is unlikely that these suffice to unravel the contribution of WNT signaling to human breast cancer. Comparing the results obtained in breast cancer cell lines to those obtained in studies with primary human breast cancer organoids and the analysis of patient-derived xenografts is warranted. Given the (epi)genetic diversity of the human breast cancer landscape, patient-to-patient heterogeneity and the interplay between breast cancer cells and their local and systemic environment, the inclusion of stromal and immune components in these experimental model systems will be essential (Holliday and Speirs, 2011; Stephens et al., 2012; Pereira et al., 2016).

## Author Contributions

RA contributed to the conception and design of the study and wrote the first draft of the manuscript. ES performed the literature survey that is summarized in Table 1 and wrote sections of the manuscript. ES and RA contributed to acquisition, analysis and interpretation of the literature. All authors contributed to the manuscript revision, read and approved the submitted version.

## Conflict of Interest

The authors declare that the research was conducted in the absence of any commercial or financial relationships that could be construed as a potential conflict of interest.

## Footnotes

1 https://jdc.jefferson.edu/cooper/
